# Carbolic Acid as an Antiseptic and Prophylactic

**Published:** 1870-08

**Authors:** 


### ARTICLE VII.

### *Carbolic Acid as an Antiseptic and Prophylactic.*

It is now certain that these putrefying (septic) germs [animal and vegetable germs floating in the air] are great causes of putrefactive *fermentation;* that fermentation is intimately connected with *inflammation;* that most *diseases* result from inflammation; that carbolic acid (antiseptic) will kill all septic germs, and thus remove many *causes* of disease; that glycerine is a very powerful healing agent; that carbolic acid is freely *soluble* in glycerine; and that their *united* application has resulted in the speedy cure of some of the most dangerous diseases. It follows, therefore, that these new therapeutic agents demand special attention.

As a rule, it is better to dissolve the crystalized carbolic acid (Calvert's) in the proportions of one part by weight of the acid to six of glycerine (*carbolate of glycerine*). In this state it can be easily diluted to any degree of strength.

In general, a *dose* of carbolic acid is 1 grain in an ounce of water.

As a *gargle*, 1 or 2 grains to an ounce of water.

As an *injection*, 1 grain to 4 ounces of water.

As a *lotion*, 15 grains to an ounce of water.

As an *ointment*, 16 grains to an ounce of benzoated lard.

As a *liniment*, 1 part to 20 of olive oil.

As a *plaster*, 1 part of carbolic acid to 3 parts of shellac.

The crystalised carbolic acid to be used as a caustic.

The carbolate of glycerine, as above, use in 1 or 2 drop doses internally.

Antiseptic oil for abscesses, 1 part of acid to 4 of boiled linseed oil.

Antiseptic putty, 6 spoonfuls of the antiseptic oil mixed with whiting.

Aqueous solution of carbolic acid is 1 part of acid to 40 of water (1 ounce of acid to a quart of hot water well agitated and filtered.)

Sick-rooms, to disinfect: place a portion of the dissolved crystals in a porcelain dish, and float it in a larger vessel of hot water.

Disinfecting purposes generally: 1 ponnd of *crystals* to 6 gallons of water. *Fluid*, 1 part to 80 of water. *Powder*, 1 ounce of crystals with 4 pounds of slaked lime.

For drains: 1 pound of the fluid carbolic acid to 5 gallons of warm water.

Tooth-ache is often cured with 1 drop of carbolate of glycerine; and diarrhœa arrested in half an hour with 2 drops in a wine glass of water.

In all cases of parasitic life it is advisable to commence with very dilute carbolate of glycerine.

Inasmuch as carbolic acid will destroy the power of *vaccine virus*, it becomes an interesting inquiry as to the possibility of using carbolic acid internally as a preventive, so as to fortify the human system against the incoming of zymotic diseases.

I have some striking facts in support of this probability; but my observation has been too limited to do more than incline to the belief; and here I leave it in the hands of the Conference, expressing my readiness to give further details if the members desire it.—*London Pharmaceutical Journal. Medical Archives.*

